# Study on the Actuating Performance of an Embedded Macro Fiber Composite Considering the Shear Lag Effect

**DOI:** 10.3390/ma15113968

**Published:** 2022-06-02

**Authors:** Jianhui Wei, Shuang Gao, Jiarui Zhang, Jianwei Tu

**Affiliations:** 1 The Second Ship Research and Institute of Wuhan, Wuhan 430064, China; wjhwxy2022110@163.com (J.W.); gshust2010@126.com (S.G.); 2 State Key Laboratory of Silicate Materials for Architectures, Wuhan University of Technology, Wuhan 430070, China

**Keywords:** embedded macro fiber composite (MFC), sandwich structure, shear lag effect, MFC actuating force formula

## Abstract

Macro fiber composite (MFC), which are new ultrathin piezoelectric smart materials, are mostly applied in the fields of shell structure deformation and vibration control. Among others, the application of embedded MFCs in sandwich structures has received wide attention. Currently, its actuating force formula is primarily acquired based on the Bernoulli–Euler Model, which does not consider the shear lag effect and actuating force of MFC ends. To study the actuating performance of an MFC in a sandwich structure, according to its action characteristics, the MFC is divided into upper and lower actuating units without any interaction between to two under the condition of plane strain, and the shear lag effect is considered between the units and the top and bottom of the sandwich structure. The actuating force of the MFC ends is obtained by considering its influence on the bending deformation of the sandwich structure, which deduces the actuating force formula of the embedded MFC. In contrast to ANSYS piezoelectric simulation, the distribution of the MFC interior normal stress is similar to the result from ANSYS piezoelectric simulation, and there is a very small deviation between the MFC end and central normal stress and the result from ANSYS piezoelectric simulation. Taking the end deflection of the sandwich structure with an embedded MFC as an example, the actuating force simulation of the MFC considering the shear lag effect is compared with the ANSYS piezoelectric simulation and actuating force simulation based on the Bernoulli–Euler model. The result indicates that the actuating force simulation of the MFC considering the shear lag effect is closer to the ANSYS piezoelectric simulation, which proves the rationality and necessity of considering the shear lag effect and end actuating force of the MFC.

## 1. Introduction

Due to its high intensity, light mass, and good plasticity, the composite sandwich structure has been widely applied in key positions under dynamic loads, such as aircraft wings, ship side structures, and engine bodies [1,2,3]. In those key positions, piezoelectric ceramics (PZT), which is the typical piezoelectric material, can be chosen to be the actuator and transducer to be embedded into the structure to reduce vibration and noise. Chen et al. [4,5] directionally embedded PZT into helicopter rotor blades to control blade vibration. Tsushima et al. [6,7] evenly embedded PZT into the aircraft wing interior to study the vibration reduction of highly flexible wing flutter by the embedded PZT and found that the embedded PZT could aptly restrain the instability of aeroelasticity and ease the gusty disturbance. Bernardini et al. [8,9] studied the method of embedding PZT into the fuselage to reduce the cabin noise and obtained an active control strategy based on a genetic algorithm and LQR algorithm. These studies show that the control methods with embedded PZT are feasible and robust. Nevertheless, its defects are not to be ignored, including weak flexibility, crisp texture, and poor application to curved surface structures [10,11]. As a result, PZT has been gradually replaced by a new piezoelectric fiber smart material—the macro fiber composite (MFC) [12], which is more applicable to sandwich structures with the advantages of strong flexibility, great output, light mass, easy installation, and long durability [13].

At present, embedded MFCs have received enormous attention in terms of vibration and deformation control of composite sandwich structures [14]. Park et al. [15] studied its inhibiting effect on the deflection of sandwich structures under pneumatic-thermal loads. A model of the structure and MFC is established through the finite element method, whose result indicates that the embedded MFC in the sandwich structure restrains the structural deflection caused by the pneumatics-thermal load better than PZT. On this basis, Park et al. [16] applied an embedded MFC further into the new active torsional blade and performed a pneumatic simulation analysis in hovering. The result shows that this active torsional rotor can effectively reduce the adverse reaction created by helicopter vibration. Tarazaga et al. [17] researched the vibration control of an embedded MFC over an inflatable stiffness cantilever and established a closed-loop control system of the positive position feedback (PPF) through the structure kinetic equation, which proves that the embedded MFC can effectively control the first-order modal vibration of the stiffness cantilever. Paradies et al. [18] found that it was effective and feasible to use embedded MFCs to control aircraft wing deformation by applying embedded MFCs to the active deformation control of composite UAV wings. Molinari et al. [19] proposed a deformation method of actuating a 3D wing based on an embedded MFC. The deformed wing can replace the traditional aileron in the numerical simulation of a light UAV with a span of 3 m, and an embedded MFC can help the deformed wing maintain very high aerodynamic efficiency. Jian et al. [20] established a composite sandwich model through the finite element method and studied the influence of quiescent voltage on the aeroelasticity stability boundary.

These studies illustrate that in the application of composite sandwich structures with embedded MFCs, most scholars consider the actuating effect of embedded MFCs on the structure from the angle of finite element modeling. Although the finite element method helps obtain a precise result for MFC modeling, it is too complicated to model and calculate using this method when a more complicated sandwich structure is analyzed. Aimed at solving a similar problem of embedded PZT, Chopra [21] used the Bernoulli–Euler Model to solve the actuating force of embedded PZT, which is easy and widely used in vibration control, deformation control, and the optimized layout of some piezoelectric materials such as PZT and MFC [22,23,24,25,26]. However, it does not consider the shear lag effect between piezoelectric material and the sandwich structure and the interaction force between piezoelectric material ends and the structure when the piezoelectric material is embedded into the sandwich structure interior, which makes it difficult to obtain the precise response of the sandwich structure. In terms of the shear lag phenomenon between the piezoelectric material and the structure, Crawley [27] proposed the uniform strain model, which can consider the shear lag effect between the piezoelectric material and structure and obtain the precise actuating force formula of the piezoelectric material. However, it is only appropriate for externally bonded piezoelectric materials and not for the direct analysis of the actuating performance of embedded MFCs.

To solve these problems, this paper mainly analyzes the shear lag effect between MFC and the structure and the actuating force of the MFC ends. First, the sandwich structure at the bottom of the embedded MFC is transformed to the equivalent plate structure with identical material parameters, based on which the zero shearing interface location of the embedded MFC is obtained through the uniform strain model, and the MFC is divided into two actuating units without interaction. Considering the shear lag effect between the MFC and the upper and lower sandwich structures, the actuating force general solution of the two MFC actuating units is calculated. Using Kirchhoff plate theory [28], the stress boundary conditions of the MFC end location and centerline location are acquired, which offers the actuating formula of the embedded MFC and analyzes the distribution of its actuating force. To verify its applicability, a composite sandwich structure with an embedded MFC is taken as an example to compare the sandwich structure deflection among the ANSYS piezoelectric simulation, Bernoulli–Euler model, and proposed MFC actuating force method in this paper.

## 2. The Constitutive Relation of MFC

**Figure 1:**
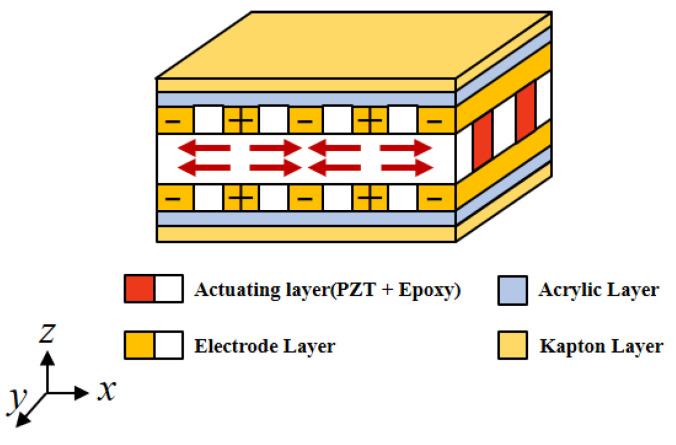
Internal structure of the MFC.

The P1 type of MFC is taken as the study object. Its internal structure and voltage direction are shown in Figure 1, where axis x is the major actuating direction and axis y is the minor direction. Under the voltage, the MFC will stretch out and draw back along the actuating direction to exert actuating force onto the structure, so the structure has stretching and bending deformation.

Due to the small thickness of the MFC, its transverse normal stress can be ignored [29], and the MFC piezoelectric constitutive relation in the xy plane is obtained as:

(1) $$\left[\begin{array}{c} \varepsilon_{x}^{m}\\ \varepsilon_{y}^{m} \end{array}\right]=\left[\begin{array}{cc} s_{11}^{m} & s_{12}^{m}\\ s_{21}^{m} & s_{22}^{m} \end{array}\right]\left[\begin{array}{c} \sigma_{x}^{m}\\ \sigma_{y}^{m} \end{array}\right]+\left[\begin{array}{c} d_{33}E_{x}\\ d_{31}E_{x} \end{array}\right]$$

(2) $$s_{11}^{m}=\frac{1}{Y_{1}^{m}},s_{12}^{m}=s_{12}^{m}=\frac{\nu_{12}^{m}}{Y_{1}^{m}}=\frac{\nu_{21}^{m}}{Y_{2}^{m}},s_{22}^{m}=\frac{1}{Y_{2}^{m}}$$

where $\varepsilon_{x}^{m}$ and $\varepsilon_{y}^{m}$ are the MFC strains along axis x and axis y, respectively; $\sigma_{x}^{m}$ and $\sigma_{y}^{m}$ are the MFC stresses along axis x and axis y; $s_{ij}^{m}$ is the MFC flexibility coefficient, i is the strain direction, and j is the stress direction; $Y_{1}^{m}$ and $Y_{2}^{m}$ are the MFC elasticity moduli along axis x and axis y; $\nu_{12}^{m}$ and $\nu_{21}^{m}$ are the MFC Poisson’s ratios; $d_{31}$ is the MFC strain produced along axis y under the unit electric field along axis x; $d_{33}$ is the MFC strain produced along axis x under the unit electric field along axis x; $E_{x}$ is the electric field intensity exerted on axis x.

The MFC actuating force MFC along axis y is too small to consider. Therefore, to study the actuating performance of embedded MFCs in the major actuating direction, the actuating performance analysis of 3D embedded MFCs is simplified to be a plane strain problem with the condition $\varepsilon_{y}=0$ [30]. Equation (1) is simplified as:

(3) $$\begin{array}{c} \varepsilon_{x}={\bar{s}}_{11}^{m}\sigma_{x}+{\bar{d}}_{33}E_{x}\\ {\bar{s}}_{11}^{m}=\frac{s_{11}^{m}s_{22}^{m}-s_{12}^{m}s_{12}^{m}}{s_{22}^{m}},{\bar{d}}_{33}=d_{33}-\frac{d_{31}s_{12}^{m}}{s_{22}^{m}} \end{array}$$

where ${\bar{s}}_{11}^{m}$ is the MFC flexibility coefficient along axis x under the plane strain condition, and ${\bar{d}}_{33}$ is the MFC piezoelectric strain coefficient under the plane strain condition.

## 3. Actuating Force Calculation of the Embedded MFC

### 3.1. Equivalent Material Parameters

**Figure 2:**
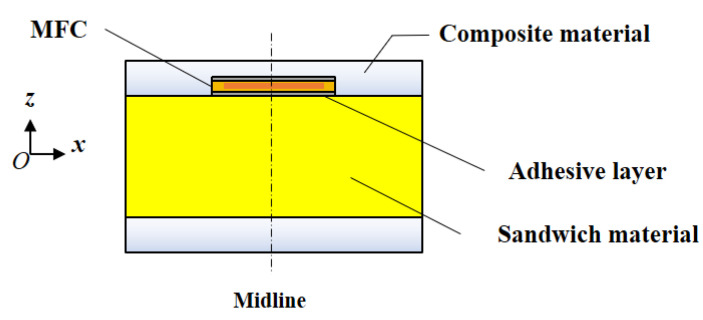
Embedded form of MFC.

The MFC embedment form and relative location in the sandwich structure are shown in Figure 2. The center of the sandwich structure with an embedded MFC is set to be the origin point of the coordinate axis. $l_{1}$ and $h_{m}$ are the valid length and thickness of the MFC actuating layer, respectively; $h_{f}$, $h_{s}$, and $h_{a}$ are the thicknesses of the composite layer, core layer, and adhesive layer, respectively.

The constitutive relations between different layers of the sandwich structure under plane strain conditions are:

(4) $$\sigma_{x}^{ft}=\frac{1}{{\bar{s}}_{11}^{f}}\varepsilon_{x}^{ft},\sigma_{x}^{s}=\frac{1}{{\bar{s}}_{11}^{s}}\varepsilon_{x}^{s},\sigma_{x}^{fb}=\frac{1}{{\bar{s}}_{11}^{f}}\varepsilon_{x}^{fb}$$

(5) $${\bar{s}}_{11}^{f}=\frac{s_{11}^{f}s_{22}^{f}-s_{12}^{f}s_{12}^{f}}{s_{22}^{f}},{\bar{s}}_{11}^{s}=\frac{s_{11}^{s}s_{22}^{s}-s_{12}^{s}s_{12}^{s}}{s_{22}^{s}}$$

(6) $$\begin{array}{l} s_{11}^{f}=\frac{1}{Y_{1}^{f}},s_{12}^{f}=s_{12}^{f}=\frac{\nu_{12}^{f}}{Y_{1}^{f}}=\frac{\nu_{21}^{f}}{Y_{2}^{f}},s_{22}^{f}=\frac{1}{Y_{2}^{f}}\\ s_{11}^{s}=\frac{1}{Y_{1}^{s}},s_{12}^{s}=s_{12}^{s}=\frac{\nu_{12}^{s}}{Y_{1}^{s}}=\frac{\nu_{21}^{s}}{Y_{2}^{s}},s_{22}^{s}=\frac{1}{Y_{2}^{s}} \end{array}$$

where $\sigma_{x}^{ft}$, $\sigma_{x}^{s}$, and $\sigma_{x}^{fb}$ are the stress of the top composite layer, core layer, and bottom composite layer along axis x; $\varepsilon_{x}^{ft}$, $\varepsilon_{x}^{s}$, and $\varepsilon_{x}^{fb}$ the strain of the top composite layer, core layer, and bottom composite layer along axis x; ${\bar{s}}_{11}^{f}$ and ${\bar{s}}_{11}^{s}$ are the flexibility coefficients of the composite material and sandwich material along axis x under the plane strain condition; $s_{ij}^{f}$ is the flexibility coefficient of composite material, i is the strain direction, and j is the stress direction; $s_{ij}^{s}$ is the flexibility coefficient of the sandwich material; i is the strain direction, and j is the stress direction; $Y_{1}^{f}$ and $Y_{2}^{f}$ are the elasticity moduli of the composite material along axis x and axis y, respectively; $Y_{1}^{s}$ and $Y_{2}^{s}$ are the elasticity moduli of sandwich material along axis x and axis y, respectively; $\nu_{12}^{f}$ and $\nu_{21}^{f}$ are Poisson’s ratios of the composite material; and $\nu_{12}^{s}$ and $\nu_{21}^{s}$ are Poisson’s ratios of the sandwich material.

**Figure 3:**
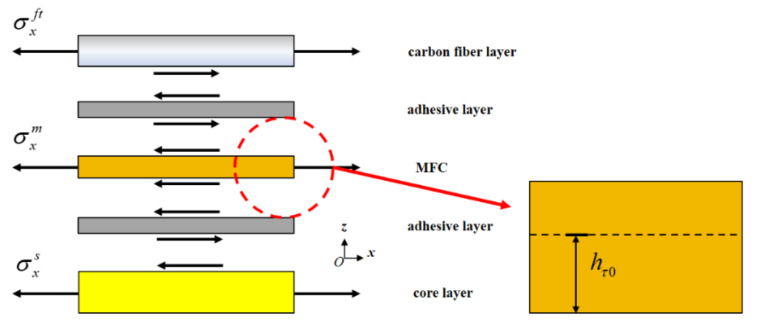
Actuating force transmission in embedded MFC.

Using the uniform strain model proposed by Crawley et al. [27] and the location of embedded MFCs in sandwich structures, it is found that MFCs, which are in the state of membrane stress, can transmit actuating force through the top and bottom adhesive layers. Due to the small elasticity modulus and thickness of the adhesive layers, it is believed to only transmit shearing stress, as shown in Figure 3 [30]. According to the transmission mode in Figure 3, the MFC has a zero shearing stress interface on a certain xy plane perpendicular to axis z. The distance between the interface and the MFC bottom surface is $h_{\tau =0}$. Based on $h_{\tau =0}$, an MFC can be divided into upper and lower actuating units without interaction, which influence the upper composite material layer and the layers in the lower MFC structure, respectively.

Due to the different material parameters of the lower MFC layers, it is inconvenient to use the uniform strain model to analyze the shear lag effect between the structure and the adhesive layer. Considering that the deformation effect of MFC on the sandwich panel structure is mainly reflected in the bending deformation, we adopt the equivalent material parameter solution method with consistent bending deformation, which requires presuming an equivalent plate structure made of homogenous material. It has equally strong bending resistance to the laminated structure at the MFC bottom. If the curvature of the laminated structure is constant, and if the same bending moment is exerted onto the laminated structure at the MFC bottom and the equivalent plate structure, the material parameters of the equivalent plate structure under the same bending deformation can be obtained. The method is as follows:

(1) Determine the bending deformation equations of the laminated structure at the MFC bottom and the equivalent plate structure, as shown in Equation (7).
  (7) $$\begin{array}{l} \varepsilon_{x,moment}^{s}=p_{x}z\;\left(-h_{s}/2\leq z\leq h_{s}/2\right)\\ \varepsilon_{x,moment}^{fb}=p_{x}z\;\left(-h_{f}-h_{s}/2\leq z\leq -h_{s}/2\right)\\ \varepsilon_{x,moment}^{eq}=p_{x}z\;\left(-h_{f}-h_{s}/2\leq z\leq h_{s}/2\right) \end{array}$$
  where $p_{x}$ is the slope of the strain distribution; $\varepsilon_{x,moment}^{s}$, $\varepsilon_{x,moment}^{fb}$, and $\varepsilon_{x,moment}^{eq}$ are the strain of the core layer, bottom composite material layer, and equivalent plate structure along axis x under the same bending deformation condition.
(2) Establish the bending moment equilibrium equations of the laminated structure at the MFC bottom and equivalent plate structure, as shown in Equation (8).
  (8) $$\begin{array}{l} \int_{-h_{s}/2}^{h_{s}/2}\sigma_{x,moment}^{s}zdz+\int_{-h_{c}/2-h_{f}}^{-h_{s}/2}\sigma_{x,moment}^{fb}zdz-M_{x}=0,\int_{-h_{c}/2-h_{f}}^{h_{s}/2}\sigma_{x,moment}^{eq}zdz-M_{x}=0\\ \sigma_{x,moment}^{s}={\bar{s}}_{11}^{s}\varepsilon_{x,moment}^{s},\sigma_{x,moment}^{fb}={\bar{s}}_{11}^{fb}\varepsilon_{x,moment}^{fb},\sigma_{x,moment}^{eq}={\bar{s}}_{11}^{eq}\varepsilon_{x,moment}^{eq} \end{array}$$
  where $\sigma_{x,moment}^{s}$, $\sigma_{x,moment}^{fb}$, and $\sigma_{x,moment}^{eq}$ are the stress of the core layer, bottom composite material layer, and equivalent plate structure along axis x under the same bending deformation condition.
(3) Combine $p_{x}$ from Equations (7) and (8) to obtain the equivalent flexibility coefficient ${\bar{s}}_{11}^{eq}$:
  (9) $${\bar{s}}_{11}^{eq}=\frac{\left(h_{f}+h_{s}\right)\left(4h_{f}^{2}+2h_{f}h_{s}+h_{s}^{2}\right){\bar{s}}_{11}^{f}{\bar{s}}_{11}^{s}}{h_{s}^{3}{\bar{s}}_{11}^{f}+4h_{f}^{3}{\bar{s}}_{11}^{s}+6h_{f}^{2}h_{s}{\bar{s}}_{11}^{s}+3h_{f}h_{s}^{2}{\bar{s}}_{11}^{s}}$$
  ${\bar{s}}_{11}^{eq}$ from (9) transforms the laminated structure at the MFC bottom to the equivalent plate structure with the identical flexibility coefficient and deduces the functional form of the shearing stress distribution of the adhesive layer at the MFC bottom through the uniform strain model.

In summary, in the MFC actuating force calculation, reasonable assumptions are listed:

(1) The MFC internal electric field is a uniform electric field along axis x. Under voltage, the MFC is in the state of membrane stress, and its actuating direction strain remains unchanged along axis x [27].
(2) Since the composite layer at the MFC top is thin, this layer is considered to be in a state of membrane stress under the MFC actuating force.
(3) The sandwich structure meets the Kirchhoff plate theory, i.e., it is believed to simultaneously create tension and compression and bending deformation, the stress along axis z is ignored, and the normal direction displacement has no relation to z [31].
(4) When the stress boundary conditions are defined for the actuating force formula of the embedded MFC, it is assumed that there is no shearing stress in each layer at the central line location of the embedded MFC; when bending deformation is produced by the MFC to the sandwich structure, the actuating performance at the MFC end is weak, which indicates that the end position only has deformation related to the overall bending.

### 3.2. Actuating Pattern of an Embedded MFC Based on the Shearing Lag Model

By combining Equation (3), Equation (9), and the uniform strain model, the MFC actuating force formula is obtained considering the shear lag model. First, according to the Kirchhoff plate theory, the strain expression of the shell structure with embedded MFC is:

(10) $$\varepsilon_{x}^{ft}\left(x,z\right)=\varepsilon_{x0}^{ft}\left(x\right),\varepsilon_{x}^{m}\left(x,z\right)=\varepsilon_{x0}^{m}\left(x\right),\varepsilon_{x}^{eq}\left(x,z\right)=\varepsilon_{x0}^{eq}\left(x\right)+zw_{,xx}^{b}\left(x\right)$$

where $\varepsilon_{x}^{eq}$ is the strain of the equivalent plate structure along axis x; $\varepsilon_{x0}^{ft}$, $\varepsilon_{x0}^{m}$, and $\varepsilon_{x0}^{eq}$ are the film strains of the upper composite layer, MFC layer, and equivalent plate layer along axis x, respectively; and $w_{,xx}^{b}$ is the slope of the strain distribution of the equivalent plate structure along axis x.

Equation (11) is obtained by substituting Equation (5) into the constitutive equations of the MFC and sandwich structure:

(11) $$\sigma_{x}^{ft}={\bar{s}}_{11}^{f}\varepsilon_{x0}^{ft},\sigma_{x}^{m}={\bar{s}}_{11}^{m}\varepsilon_{x}^{m},\sigma_{x}^{eq}={\bar{s}}_{11}^{eq}\varepsilon_{x}^{eq}={\bar{s}}_{11}^{eq}\left(\varepsilon_{x0}^{eq}+zw_{,xx}^{b}\right)$$

where $\sigma_{x}^{ft}$, $\sigma_{x}^{m}$, and $\sigma_{x}^{eq}$ are the stresses of the upper composite layer, MFC layer, and equivalent plate layer along axis x, respectively.

The axial force and bending moment of the upper composite layer and equivalent plate layer are:

(12) $$N_{x}^{ft}=\int_{\frac{h_{s}+h_{f}}{2}+2h_{b}+h_{m}}^{\frac{h_{s}+h_{f}}{2}+2h_{b}+h_{m}+h_{f}}\sigma_{x}^{ft}dz,N_{x}^{eq}=\int_{-\frac{h_{s}+h_{f}}{2}-h_{f}}^{\frac{h_{s}+h_{f}}{2}}\sigma_{x}^{eq}dz$$

(13) $$M_{x}^{eq}=\int_{-\frac{h_{s}+h_{f}}{2}-h_{f}}^{\frac{h_{s}+h_{f}}{2}}\sigma_{x}^{eq}zdz$$

where $N_{x}^{t}$ and $N_{x}^{b}$ are the axial forces of the upper composite layer and equivalent plate layer along axis x; $M_{x}^{b}$ is the bending moment of the equivalent plate structure along axis x.

Since the deformation effect of MFC on the structure is provided by the interface shear stress between MFC and the structure, considering the shearing stress on the interface between the MFC and the upper composite layer and equivalent plate layer, the mechanical equilibrium equation is obtained:

(14) $$\frac{dN_{x}^{ft}}{dx}+\tau_{x}^{ft}=0,\frac{dN_{x}^{eq}}{dx}+\tau_{x}^{eq}=0,\frac{dM_{x}^{eq}}{dx}+\frac{\tau_{x}^{eq}\left(h_{s}+h_{f}\right)}{2}=0$$

where $\tau_{x}^{ft}$ and $\tau_{x}^{eq}$ are the shearing stress of the upper composite layer and laminated structure at the MFC bottom along axis x, respectively, and they are also the actuating shearing stress of MFC for the structure.

According to Equations (12)–(14), the stress equilibrium equation is obtained on the MFC interface between the upper composite layer and the laminated layer at the MFC bottom:

(15) $$\begin{array}{l} \frac{d}{dx}\sigma_{x}^{ft}+\frac{\tau_{x}^{ft}}{h_{f}}=0,\frac{d}{dx}\sigma_{x1}^{eq}+\frac{4\tau_{x}^{eq}}{h_{s}+h_{f}}=0\\ \left(h_{m}-h_{\tau =0}\right)\frac{d}{dx}\sigma_{x}^{m}-\tau_{x}^{ft}=0,h_{\tau =0}\frac{d}{dx}\sigma_{x}^{m}-\tau_{x}^{eq}=0\\ \sigma_{x1}^{eq}={\sigma_{x}^{eq}|}_{z=\frac{h_{s}+h_{f}}{2}} \end{array}$$

where $\sigma_{x}^{ft}$ is the stress of the upper composite layer along axis x, and $\sigma_{x1}^{eq}$ is the stress of the equivalent plate layer along axis x at $z=0.5\left(h_{s}+h_{f}\right)$.

Considering the shearing deformation of the adhesive layer, $\tau_{x}^{ft}$ and $\tau_{x}^{eq}$ can be expressed as:

(16) $$\tau_{x}^{ft}=\frac{G_{a}}{h_{a}}\left(u^{m}-u^{ft}\right),\tau_{x}^{eq}=\frac{G_{a}}{h_{a}}\left(u^{m}-u_{1}^{eq}\right),u_{1}^{eq}={u^{eq}|}_{z=\frac{h_{s}+h_{f}}{2}}$$

where $u_{1}^{eq}$ is the displacement of the equivalent plate structure at $z=0.5\left(h_{s}+h_{f}\right)$.

This is obtained by combining Equations (11), (15), and (16):

(17) $$\frac{1}{{\bar{s}}_{11}^{f}}\frac{d^{2}}{dx^{2}}\varepsilon_{x}^{ft}+\frac{G_{a}}{h_{a}h_{f}}\left(\varepsilon_{x}^{m}-\varepsilon_{x}^{ft}\right)=0,\frac{h_{m}-h_{\tau =0}}{{\bar{s}}_{11}^{m}}\frac{d^{2}}{dx^{2}}\varepsilon_{x}^{m}-\frac{G_{a}}{h_{a}}\left(\varepsilon_{x}^{m}-\varepsilon_{x}^{ft}\right)=0$$

(18) $$\frac{1}{{\bar{s}}_{11}^{eq}}\frac{d^{2}}{dx^{2}}\varepsilon_{x1}^{eq}+\frac{4G_{a}}{h_{a}\left(h_{s}+h_{f}\right)}\left(\varepsilon_{x}^{m}-\varepsilon_{x1}^{eq}\right)=0,\frac{h_{\tau =0}}{{\bar{s}}_{11}^{m}}\frac{d^{2}}{dx^{2}}\varepsilon_{x}^{m}-\frac{G_{a}}{h_{a}}\left(\varepsilon_{x}^{m}-\varepsilon_{x1}^{eq}\right)=0,\varepsilon_{x1}^{eq}={\varepsilon_{x1}^{eq}|}_{z=\frac{h_{s}+h_{f}}{2}}$$

This is obtained by organizing Equations (17) and (18):

(19) $$\frac{d^{4}}{dx^{4}}\varepsilon_{x}^{ft}-\alpha^{ft}\frac{d^{2}}{dx^{2}}\varepsilon_{x}^{ft}=0,\frac{d^{4}}{dx^{4}}\varepsilon_{x1}^{eq}-\alpha^{eq}\frac{d^{2}}{dx^{2}}\varepsilon_{x1}^{eq}=0$$

(20) $$\alpha^{ft}=\sqrt{\frac{G_{a}{\bar{s}}_{11}^{f}}{h_{a}h_{f}}+\frac{{\bar{s}}_{11}^{m}G_{a}}{h_{a}\left(h_{m}-h_{\tau =0}\right)}},\alpha^{eq}=\sqrt{\frac{4G_{a}{\bar{s}}_{11}^{eq}}{h_{a}\left(h_{f}+h_{s}\right)}+\frac{{\bar{s}}_{11}^{m}G_{a}}{h_{a}h_{\tau =0}}}$$

Under voltage, MFC is always in the state of membrane stress, and the two MFC actuating units, whose dividing line is the zero shearing stress interface, have equal normal stress, i.e., the MFC upper and lower adhesive layers have identical shearing stress function distributions. Therefore, Equations (19) and (20) are combined to calculate the relative position of the zero shearing stress interface $h_{\tau =0}$, which is shown in Equation (21).

(21) $$\frac{G_{a}{\bar{s}}_{11}^{f}}{h_{a}h_{f}}+\frac{{\bar{s}}_{11}^{m}G_{a}}{h_{a}\left(h_{m}-h_{\tau =0}\right)}-\left(\frac{4G_{a}{\bar{s}}_{11}^{eq}}{h_{a}\left(h_{f}+h_{s}\right)}+\frac{{\bar{s}}_{11}^{m}G_{a}}{h_{a}h_{\tau =0}}\right)=0$$

This is obtained by solving Equation (21):

(22) $$h_{\tau =0}=\frac{h_{m}}{2}+\beta \pm \frac{\sqrt{h_{m}^{2}+4\beta^{2}}}{2},\beta =\frac{h_{f}\left(h_{s}+h_{f}\right){\bar{s}}_{11}^{m}}{h_{s}{\bar{s}}_{11}^{f}+h_{f}\left(-4{\bar{s}}_{11}^{eq}+{\bar{s}}_{11}^{f}\right)}$$

When $\beta <-h_{m}/2$, $h_{\tau =0}$ is less than 0 or less than $\sqrt{2}h_{m}/2$ and more than 0; when $-h_{m}/2<\beta <0$, $h_{\tau =0}$ is more than $h_{m}$ or less than $\left(2-\sqrt{2}\right)h_{m}/4$ and more than $\sqrt{2}h_{m}/8$; when $0<\beta <h_{m}/2$, $h_{\tau =0}$ is more than $h_{m}$ or less than $\left(1-3\sqrt{2}/8\right)h_{m}$ and more than 0; when $\beta >h_{m}/2$, $h_{\tau =0}$ is more than $h_{m}$ or less than $\left(1-\sqrt{2}/2\right)h_{m}$ and more than 0. Therefore, in the MFC interior, there is always a unique and proper zero shearing stress interface $h_{\tau =0}$ as the dividing line between the upper and lower MFC actuating units. According to Equations (19), (20) and (22), the general solution of the internal force formula of the upper and lower MFC actuating units is obtained as follows:

(23) $$\sigma_{x}^{m}=C_{1}+C_{2}\cosh\left(\alpha^{ft}x\right)=C_{1}+C_{2}\cosh\left(\alpha^{eq}x\right)$$

where $C_{1}$ and $C_{2}$ are the undetermined coefficients of the actuating force formula related to the stress boundary condition.

Since the embedded MFC is located inside the sandwich structure, its ends create an interaction force with the sandwich structure. Therefore, it is necessary to consider not only the stress boundary conditions at the centerline position of MFC, but also the stress boundary conditions at the end position. Firstly, the stress boundary conditions at the centerline position of MFC are calculated. According to the shell structure theory concerning shearing deformation [31,32,33,34], there is no shearing deformation at the centerline of the sandwich plate with an embedded MFC. Considering the stress equilibrium condition and deformation compatibility condition from the Kirchhoff plate theory, the strain distribution equation, stress equilibrium equation, and deformation compatibility equation at the centerline can be defined as:

(24) $$\varepsilon_{xc}^{ft}=q_{x1}^{t},\varepsilon_{xc}^{m}=q_{x1}^{m},\varepsilon_{xc}^{eq}=p_{x1}^{eq}z+q_{x1}^{eq}$$

(25) $$\begin{array}{l} \int_{\frac{h_{s}}{2}+h_{m}+2h_{a}}^{\frac{h_{s}}{2}+h_{m}+2h_{a}+h_{f}}\frac{1}{{\bar{s}}_{11}^{f}}q_{x1}^{t}dz+\int_{\frac{h_{s}}{2}+h_{a}}^{\frac{h_{s}}{2}+h_{m}+h_{a}}\frac{1}{{\bar{s}}_{11}^{m}}\left(q_{x1}^{m}-{\bar{d}}_{33}E_{x}\right)dz+\int_{-\frac{h_{s}}{2}-h_{f}}^{\frac{h_{s}}{2}}\frac{1}{{\bar{s}}_{11}^{eq}}\left(p_{x1}^{eq}z+q_{x1}^{eq}\right)dz=0\\ \int_{\frac{h_{s}}{2}+h_{m}+2h_{a}}^{\frac{h_{s}}{2}+h_{m}+2h_{a}+h_{f}}\frac{1}{{\bar{s}}_{11}^{f}}q_{x1}^{t}zdz+\int_{\frac{h_{s}}{2}+h_{a}}^{\frac{h_{s}}{2}+h_{m}+h_{a}}\frac{1}{{\bar{s}}_{11}^{m}}\left(q_{x1}^{m}-{\bar{d}}_{33}E_{x}\right)zdz+\int_{-\frac{h_{s}}{2}-h_{f}}^{\frac{h_{s}}{2}}\frac{1}{{\bar{s}}_{11}^{eq}}\left(p_{x1}^{eq}z+q_{x1}^{eq}\right)zdz=0\\ \int_{\frac{h_{s}}{2}+h_{m}+2h_{a}}^{\frac{h_{s}}{2}+h_{m}+2h_{a}+h_{f}}\frac{1}{{\bar{s}}_{11}^{f}}q_{x1}^{t}dz+\int_{\frac{h_{s}}{2}+h_{a}+h_{\tau =0}}^{\frac{h_{s}}{2}+h_{m}+h_{a}}\frac{1}{{\bar{s}}_{11}^{m}}\left(q_{x1}^{m}-{\bar{d}}_{33}E_{x}\right)dz=0\\ q_{x1}^{m}=\frac{p_{x1}^{eq}h_{s}}{2}+q_{x1}^{eq} \end{array}$$

where $\varepsilon_{xc}^{ft}$, $\varepsilon_{xc}^{m}$, and $\varepsilon_{xc}^{eq}$ are the strain of the upper composite layer, MFC layer, and equivalent plate layer at the centerline, respectively; $q_{x1}^{t}$, $q_{x1}^{m}$, and $q_{x1}^{eq}$ are the membrane strain of the upper composite layer, MFC layer, and equivalent plate layer at the centerline, respectively; $p_{x1}^{eq}$ is the slope of the strain distribution of the equivalent plate structure at the centerline.

This is obtained by solving Equation (25):

(26) $$q_{x1}^{t}=\frac{{\bar{s}}_{11}^{f}\left(h_{m}-h_{\tau =0}\right){\left(h_{s}+h_{f}\right)}^{2}{\bar{d}}_{33}E_{x}}{h_{f}\left(h_{\tau =0}{\bar{s}}_{11}^{eq}\left(12h_{a}+4h_{s}+7h_{f}+6h_{m}\right)-6h_{a}h_{m}{\bar{s}}_{11}^{eq}+h_{s}^{2}{\bar{s}}_{11}^{m}+2h_{s}h_{f}{\bar{s}}_{11}^{m}+h_{f}^{2}{\bar{s}}_{11}^{m}-3h_{f}h_{m}{\bar{s}}_{11}^{eq}-3h_{m}^{2}{\bar{s}}_{11}^{eq}\right)}$$

(27) $$q_{x1}^{m}=\frac{{\bar{d}}_{33}E_{x}{\bar{s}}_{11}^{eq}\left(h_{\tau =0}\left(12h_{a}+4h_{s}+7h_{f}+6h_{m}\right)-3h_{m}\left(2h_{a}+h_{f}+h_{m}\right)\right)}{h_{\tau =0}{\bar{s}}_{11}^{eq}\left(12h_{a}+4h_{s}+7h_{f}+6h_{m}\right)-6h_{a}h_{m}{\bar{s}}_{11}^{eq}+h_{s}^{2}{\bar{s}}_{11}^{m}+2h_{s}h_{f}{\bar{s}}_{11}^{m}+h_{f}^{2}{\bar{s}}_{11}^{m}-3h_{f}h_{m}{\bar{s}}_{11}^{eq}-3h_{m}^{2}{\bar{s}}_{11}^{eq}}$$

(28) $$q_{x1}^{eq}=\frac{{\bar{d}}_{33}E_{x}{\bar{s}}_{11}^{eq}\left(h_{\tau =0}\left(h_{f}\left(12h_{a}+4h_{s}+6h_{m}\right)+h_{s}^{2}+5h_{s}h_{f}\right)-3h_{f}h_{m}\left(2h_{a}+h_{f}+h_{m}\right)\right)}{\left(h_{s}+h_{f}\right)\left(h_{\tau =0}{\bar{s}}_{11}^{eq}\left(12h_{a}+4h_{s}+7h_{f}+6h_{m}\right)-6h_{a}h_{m}{\bar{s}}_{11}^{eq}+h_{s}^{2}{\bar{s}}_{11}^{m}+2h_{s}h_{f}{\bar{s}}_{11}^{m}+h_{f}^{2}{\bar{s}}_{11}^{m}-3h_{f}h_{m}{\bar{s}}_{11}^{eq}-3h_{m}^{2}{\bar{s}}_{11}^{eq}\right)}$$

(29) $$p_{x1}^{eq}=\frac{6{\bar{d}}_{33}E_{x}{\bar{s}}_{11}^{eq}\left(h_{\tau =0}\left(4h_{a}+h_{s}+2\left(h_{f}+h_{m}\right)\right)-h_{m}\left(2h_{a}+h_{f}+h_{m}\right)\right)}{\left(h_{s}+h_{f}\right)\left(h_{\tau =0}{\bar{s}}_{11}^{eq}\left(12h_{a}+4h_{s}+7h_{f}+6h_{m}\right)-6h_{a}h_{m}{\bar{s}}_{11}^{eq}+h_{s}^{2}{\bar{s}}_{11}^{m}+2h_{s}h_{f}{\bar{s}}_{11}^{m}+h_{f}^{2}{\bar{s}}_{11}^{m}-3h_{f}h_{m}{\bar{s}}_{11}^{eq}-3h_{m}^{2}{\bar{s}}_{11}^{eq}\right)}$$

With regard to the location of MFC ends, there is deformation related only to the overall bending of the sandwich structure. The strain distribution equation at the ends is defined as:

(30) $$\begin{array}{l} \varepsilon_{xe}^{ft}=p_{x2}^{eq}z\;\left(h_{s}/2+2h_{a}+h_{m}\leq z\leq h_{s}/2+2h_{a}+h_{m}+h_{f}\right)\\ \varepsilon_{xe}^{eq}=p_{x2}^{eq}z\;\left(-h_{f}-h_{s}/2\leq z\leq h_{s}/2\right) \end{array}$$

where $\varepsilon_{xe}^{ft}$, $\varepsilon_{xe}^{m}$, and $\varepsilon_{xe}^{eq}$ are the strain of the upper composite layer, MFC layer, and equivalent plate layer at the ends, respectively; $p_{x2}^{eq}$ is the slope of the strain distribution of the equivalent plate structure at the ends.

Since there is warpage deformation at the ends and greater shearing stress along the thickness direction, it is suitable to establish the stress equilibrium equation at the ends based on the condition of the bending moment equilibrium:

(31) $$\int_{\frac{h_{s}}{2}+h_{a}}^{\frac{h_{s}}{2}+h_{a}+h_{m}}{\bar{\sigma }}_{xe}^{m}zdz+\int_{\frac{h_{s}}{2}+2h_{a}+h_{m}}^{\frac{h_{s}}{2}+2h_{a}+h_{m}+h_{f}}{\bar{s}}_{11}^{eq}p_{x2}^{eq}z^{2}dz+\int_{-\frac{h_{s}}{2}-h_{f}}^{\frac{h_{s}}{2}}{\bar{s}}_{11}^{eq}p_{x2}^{eq}z^{2}dz=0$$

where ${\bar{\sigma }}_{xe}^{m}$ is the actuating stress at the ends of the MFC layer.

This is obtained by solving Equation (31):

(32) $${\bar{\sigma }}_{xe}^{m}=\frac{p_{x2}^{eq}\left(\begin{array}{l} 48h_{a}^{2}h_{m}{\bar{s}}_{11}^{eq}+24h_{a}h_{f}{\bar{s}}_{11}^{eq}\left(h_{s}+h_{f}+2h_{m}\right)+h_{s}^{3}{\bar{s}}_{11}^{f}+\\ 3h_{s}^{2}h_{f}\left({\bar{s}}_{11}^{eq}+{\bar{s}}_{11}^{f}\right)+6h_{s}h_{f}\left(h_{f}\left({\bar{s}}_{11}^{eq}+{\bar{s}}_{11}^{f}\right)+2h_{m}{\bar{s}}_{11}^{eq}\right)+\\ 4h_{f}\left(h_{f}^{2}\left({\bar{s}}_{11}^{eq}+{\bar{s}}_{11}^{f}\right)+3h_{f}h_{m}{\bar{s}}_{11}^{eq}+3h_{m}^{2}{\bar{s}}_{11}^{eq}\right) \end{array}\right)}{6h_{m}{\bar{s}}_{11}^{eq}{\bar{s}}_{11}^{f}\left(2h_{a}+h_{s}+h_{m}\right)}$$

The undetermined coefficient equation set is established by substituting Equations (26)–(29) and (32) into Equation (23):

(33) $$C_{1}+C_{2}=\frac{1}{{\bar{s}}_{11}^{m}}\left(q_{x1}^{m}-{\bar{d}}_{33}E_{x}\right),C_{1}+C_{2}\cosh\left(\frac{\alpha^{ft}l_{1}}{2}\right)={\bar{\sigma }}_{xe}^{m}$$

Solving Equation (33) yields:

(34) $$C_{1}=\frac{\frac{1}{{\bar{s}}_{11}^{m}}\left(q_{x1}^{m}-{\bar{d}}_{33}E_{x}\right)\cosh\left(\frac{\alpha^{ft}l_{1}}{2}\right)-{\bar{\sigma }}_{xe}^{m}}{\cosh\left(\frac{\alpha^{ft}l_{1}}{2}\right)-1},C_{2}=\frac{{\bar{\sigma }}_{xe}^{m}-\frac{1}{{\bar{s}}_{11}^{m}}\left(q_{x1}^{m}-{\bar{d}}_{33}E_{x}\right)}{\cosh\left(\frac{\alpha^{ft}l_{1}}{2}\right)-1}$$

Substituting Equation (34) into Equation (23) yields:

(35) $$\begin{array}{cc} \sigma_{x}^{m} & =\frac{\frac{1}{{\bar{s}}_{11}^{m}}\left(q_{x1}^{m}-{\bar{d}}_{33}E_{x}\right)\cosh\left(\frac{\alpha^{ft}l_{1}}{2}\right)-{\tilde{\sigma }}_{xe}^{m}}{\cosh\left(\frac{\alpha^{ft}l_{1}}{2}\right)-1}+\frac{{\tilde{\sigma }}_{xe}^{m}-\frac{1}{{\bar{s}}_{11}^{m}}\left(q_{x1}^{m}-{\bar{d}}_{33}E_{x}\right)}{\cosh\left(\frac{\alpha^{ft}l_{1}}{2}\right)-1}\cosh\left(\alpha^{ft}x\right)\\ & =\frac{\frac{1}{{\bar{s}}_{11}^{m}}\left(q_{x1}^{m}-{\bar{d}}_{33}E_{x}\right)\cosh\left(\frac{\alpha^{eq}l_{1}}{2}\right)-{\tilde{\sigma }}_{xe}^{m}}{\cosh\left(\frac{\alpha^{eq}l_{1}}{2}\right)-1}+\frac{{\tilde{\sigma }}_{xe}^{m}-\frac{1}{{\bar{s}}_{11}^{m}}\left(q_{x1}^{m}-{\bar{d}}_{33}E_{x}\right)}{\cosh\left(\frac{\alpha^{eq}l_{1}}{2}\right)-1}\cosh\left(\alpha^{eq}x\right) \end{array}$$

The actuating shearing stresses $\tau_{x}^{ft}$ and $\tau_{x}^{eq}$ are obtained by combining Equations (15) and (35):

(36) $$\tau_{x}^{ft}=\frac{\alpha^{ft}\left(h_{m}-h_{\tau =0}\right)\left({\bar{\sigma }}_{xe}^{m}-\frac{1}{{\bar{s}}_{11}^{m}}\left(q_{x1}^{m}-{\bar{d}}_{33}E_{x}\right)\right)}{\cosh\left(\frac{\alpha^{ft}l_{1}}{2}\right)-1}\sinh\left(\alpha^{ft}x\right)$$

(37) $$\tau_{x}^{eq}=\frac{\alpha^{eq}h_{\tau =0}\left({\bar{\sigma }}_{xe}^{m}-\frac{1}{{\bar{s}}_{11}^{m}}\left(q_{x1}^{m}-{\bar{d}}_{33}E_{x}\right)\right)}{\cosh\left(\frac{\alpha^{eq}l_{1}}{2}\right)-1}\sinh\left(\alpha^{eq}x\right)$$

## 4. Analysis of Examples

By comparing the normal stress of the MFC, stress at the centerline, and actuating stress at the ends, we verify that the above formulas are applicable. The sandwich structure, core board, size, and material properties of the MFC, which are identical to those in Figure 2, are shown in Table 1.

**Figure 4:**
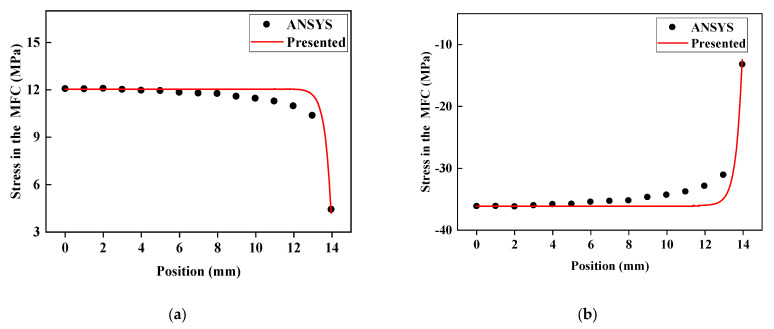
Normal stress distribution of MFC along the actuating direction: (**a**) −500 V; (**b**) 1500 V.

**Figure 5:**
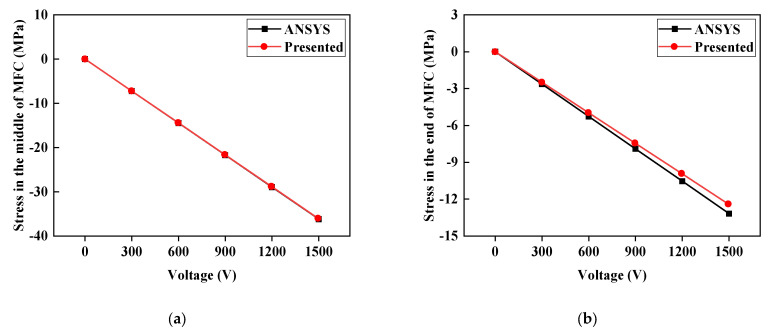
Relationship between voltage and stress in the MFC: (**a**) Stress in the middle of the MFC; (**b**) stress at the end of the MFC.

By applying voltage to the MFC, we compare the normal stress distributions of the embedded MFC under −500 V and 1500 V along the major actuating direction and the deviation of the theoretically calculated value of the stress at the MFC centerline and MFC ends under different voltages from the ANSYS piezoelectric simulation value, as shown in Figure 4 and Figure 5.

It can be seen from Figure 4 and Figure 5 that the distribution trend of normal stress in MFC is consistent with the finite element results of ANSYS. Both are smooth and steady in the middle part and have sudden stress changes at the ends. Especially in Figure 4, between the middle and end of MFC, there is a phenomenon whereby the stress calculated by ANSYS is less than the stress calculated by the formula in this paper. This is because, in the ANSYS piezoelectric finite element simulation, the piezoelectric strain constant $d_{31}$ in the thickness direction is considered to reduce the normal stress in the main actuating direction. Since the normal stress in the middle of MFC is the largest, it is less affected by $d_{31}$ in the thickness direction. Near the end position, the normal stress of MFC is small, and the influence of $d_{31}$ appears. For the end, the formula proposed in this paper can describe the stress state of the end through the boundary conditions, so the error between the middle and end stress results is small. In Figure 5, the reason the middle normal stress is more accurate than the end normal stress is that the stress state of the middle position is simple. Assuming that the strain is linearly distributed along the thickness direction, accurate results can be obtained. The material properties of the end position along the actuating direction have a transition, and the assumption that the strain distribution along the thickness direction is linear causes the calculation results to have some errors. The theoretically calculated values of stress at the centerline and the ends greatly coincide with the ANSYS simulation result. The maximum deviation is 5.61%, and the minimum is 0.2%. The comparison of the stress at the MFC ends demonstrates an interaction force between MFC ends and sandwich structure, which is too strong to ignore and accounts for the necessity of considering the actuating force at the MFC ends.

**Figure 6:**
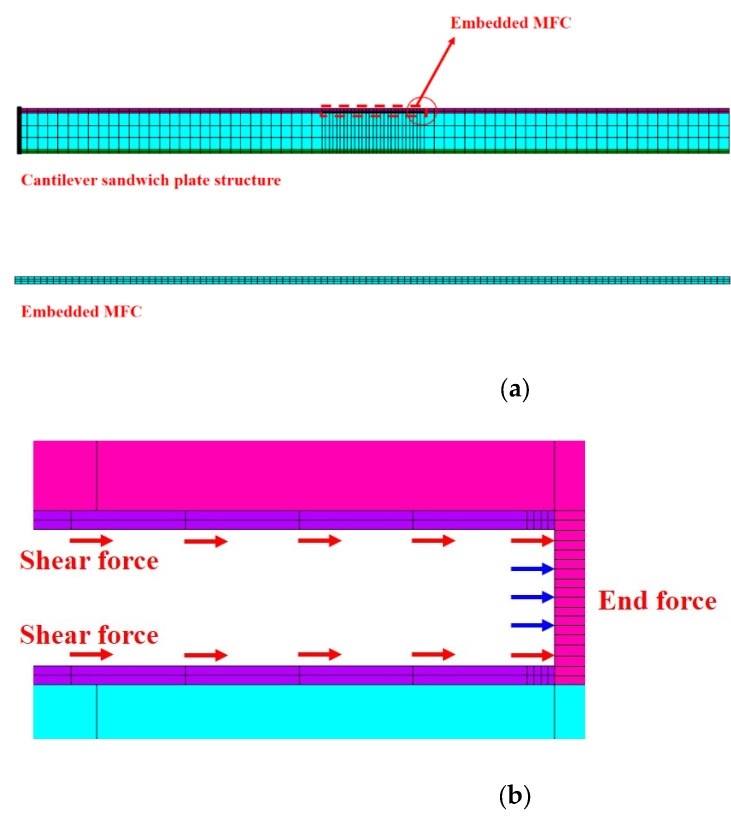
ANSYS finite element model of the sandwich plate structure with embedded MFC: (**a**) Finite element model; (**b**) action form of actuating stress.

Taking the end deflection comparison of the one-dimensional composite sandwich structure with embedded MFC as an example, the ANSYS piezoelectric simulation analysis is further accomplished under a voltage of 0–1500 V. The proposed method is compared with the Bernoulli–Euler model [21]. The finite element model of the composite sandwich structure is shown in Figure 6. The material parameters of the P1 MFC and core board are listed in Table 1.

**Figure 7:**
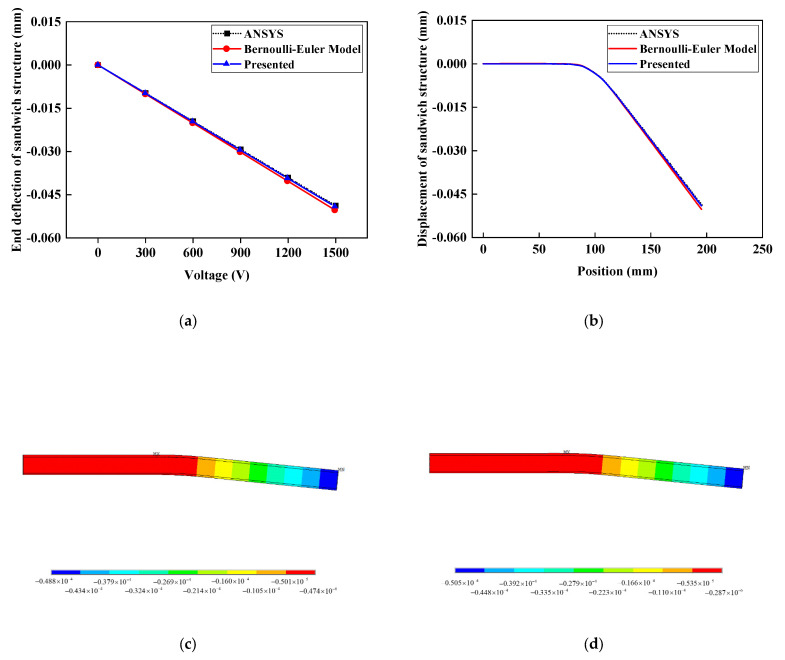

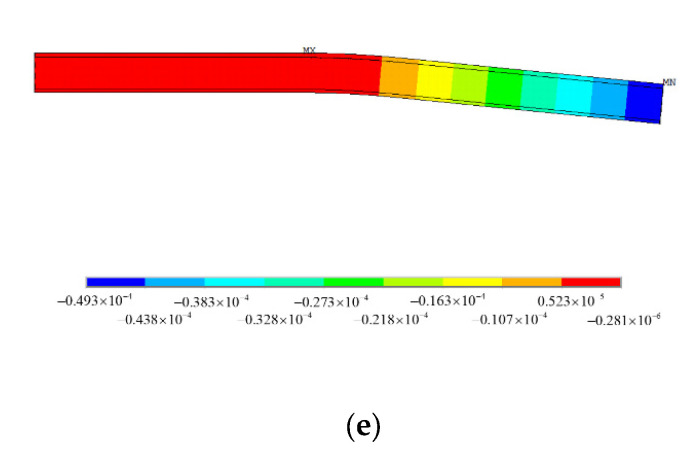
Relationship between voltage and structural displacement: (**a**) End deflection under different voltages; (**b**) structure displacement curve under 1500 V; (**c**) displacement contour map based on piezoelectric simulation under 1500 V; (**d**) displacement contour map based on Bernoulli–Euler model under 1500 V; (**e**) displacement contour map based on the presented method under 1500 V.

In the ANSYS piezoelectric analysis, the PLANE13 unit is adopted to simulate MFC, and the PLANE182 unit is adopted to simulate the adhesive layer, composite layer, and core layer. Because of the great shearing stress at the MFC ends, the adhesive layer at the MFC ends is locally refined during the modeling process to improve the precision of the simulation [37,38]. By applying voltage to the MFC with voltage constraint conditions, the ANSYS piezoelectric analysis result is obtained under plane strain with respect to the thickness. In the actuating force simulation in this paper, Equations (35)–(37) are used to calculate the actuating force at the MFC ends and actuating shearing force of MFC to the upper composite layer and lower structure. From Figure 4 and Figure 6 and Equation (35), the MFC actuating force is mostly exerted around the MFC ends, so the actuating force should be exerted on the sandwich structure according to the actuating force distribution in Figure 6b. When the actuating force is exerted, the MFC model in the ANSYS piezoelectric analysis must be removed, the end actuating force should be exerted in the form of a linear load, and the actuating shearing force should be exerted on every node on the upper and lower surfaces of the ends. In the actuating force simulation of the Bernoulli–Euler Model, the MFC actuating bending moment must be exerted on the node on the most lateral side of the ends. The results of ANSYS piezoelectric simulation, the actuating force simulation under different voltages, and the displacement contour map of ANSYS simulation under 1500 V voltage are shown in Figure 7.

From the above end displacement, deflection curve, and deflection contour map, it can be seen that the actuating force displacement result of the embedded MFC is more consistent with the ANSYS piezoelectric simulation result. Under different voltages, the end displacement deviation by the method in the paper is only 1.31%, whereas the deviation based on the Bernoulli–Euler model is as great as 3.5%. Because the Bernoulli–Euler model does not consider the end actuating force, the actuating force between the MFC layer and upper composite layer is enhanced, and the actuating bending moment increases, which leads to a greater calculation result. Thus, the calculation results of the method in the paper are more accurate.

## 5. Conclusions

This paper deduces an actuating force formula of an embedded MFC considering the shearing lag effect and compares the ANSYS piezoelectric simulation and actuating force simulation of the composite sandwich structure. The following conclusions are drawn:

(1) A research idea of investigating the actuating performance of an embedded MFC is proposed, which divides MFC into two non-interaction actuating units, and the laminated structure of the lower part of MFC is equivalent to the plate structure composed of homogenous material, which simplifies the analysis of the shear lag effect.
(2) In the composite sandwich structure with an embedded MFC, there is shearing lag on the upper and lower surfaces of the MFC, and the MFC end actuating force is too great to ignore. The internal force calculation result of the embedded MFC actuating force formula is highly consistent with the ANSYS piezoelectric simulation.
(3) The simulation of the composite sandwich structure was compared with Bernoulli–Euler model and shows that the deflection calculation result of the MFC actuating force considering the shearing lag effect is closer to that of ANSYS piezoelectric simulation. Thus, consideration of the MFC end actuating force is reasonable and applicable. This method can be further promoted to the fields of vibration reduction and deformation control of composite sandwich structures.

## Figures and Tables

**Table 1:** Material parameters of the MFC and sandwich plate structure.

Material Parameters	Composite Sandwich Structure			MFC Types
	Composite [35]	Core Board [36,37]	Adhesive Layer [30]	P1 Type [31]
Elasticity modulus	10.4 GPa	2.45 Gpa	4.7 Gpa	30.336/15.857 Gpa (axis x/axis z)
Shear modulus	6.20 Gpa	0.94 Gpa	1.67 Gpa	6.06 Gpa
Poisson ratio	0.2	0.3	0.4	0.31
Piezoelectric strain constant $d_{33}$	/	/	/	460 × 10^−12^ C/N
Piezoelectric strain constant $d_{31}$	/	/	/	−210 × 10^−12^ C/N
Geometric size	196 × 42 × 1 mm^2^	196 × 42 × 10 mm^2^	28 × 14 × 0.04 mm^2^	28 × 14 × 0.3 mm^2^

## Data Availability

The data presented in this study are available on request from the corresponding author.
